# Laparoscopic Removal of a Fish Bone Causing an Abdominal Wall Abscess Using Laparoscopic Ultrasonography: A Case Report

**DOI:** 10.70352/scrj.cr.26-0118

**Published:** 2026-06-17

**Authors:** Ippei Uezu, Yoei Takamiyagi, Tomofumi Chibana, Koji Kato, Yoshiki Chinen, Masayoshi Nagahama, Hirofumi Tomori

**Affiliations:** Department of Surgery, Naha City Hospital, Naha, Okinawa, Japan

**Keywords:** abdominal wall abscess, fish bone, laparoscopic ultrasonography

## Abstract

**INTRODUCTION:**

Abdominal wall abscess caused by gastrointestinal penetration of an ingested fish bone is rare and often difficult to diagnose and manage. We report an educational case highlighting staged management and the practical use of laparoscopic ultrasonography for safe removal of a retained foreign body.

**CASE PRESENTATION:**

A 57-year-old Japanese woman presented with recurrent right lower quadrant abdominal pain 1 month after spontaneous resolution of an initial episode. CT revealed inflammatory changes around the transverse colon, a contiguous abdominal wall abscess, and a linear calcified foreign body consistent with a fish bone. Initial conservative treatment with intravenous antibiotics resulted in symptom relief and marked reduction of the abscess; however, the foreign body persisted on follow-up imaging. Elective laparoscopic surgery was therefore performed. Because direct visual identification was difficult due to inflammatory adhesions, laparoscopic ultrasonography was used intraoperatively to localize and safely remove the fish bone without bowel resection. The operative time was 89 min, blood loss was minimal, and the postoperative course was uneventful.

**CONCLUSIONS:**

Staged management combined with laparoscopic ultrasonography is a useful and minimally invasive approach for treating abdominal wall abscesses caused by ingested foreign bodies when intraoperative visual identification is challenging.

## INTRODUCTION

Most gastrointestinal foreign bodies are excreted spontaneously, and complications such as perforation or penetration of the gastrointestinal tract occur in fewer than 1% of cases. In rare situations, however, these complications can lead to peritonitis or localized abscess formation. Fish bones are a common cause of gastrointestinal perforation in regions with high fish consumption, whereas abdominal wall abscess formation secondary to fish bone penetration remains extremely rare. Herein, we report a rare case of an abdominal wall abscess caused by penetration of the transverse colon by a fish bone. The patient was successfully managed by staged treatment with initial conservative therapy followed by elective laparoscopic removal. This case highlights the technical value of laparoscopic ultrasonography for accurate localization and safe removal of retained foreign bodies when visual identification is challenging.

## CASE PRESENTATION

A 57-year-old Japanese woman experienced severe right lower quadrant abdominal pain 1 month before presentation, which resolved spontaneously within approximately 1 week. She subsequently developed recurrent episodes of right lower quadrant abdominal pain and consulted a local physician, who suspected acute appendicitis and referred her to our hospital. Her medical history was notable only for hypertension.

On admission, she was afebrile and had tenderness in the right lower quadrant without muscular guarding. Laboratory examination revealed an elevated C-reactive protein level of 3.98 mg/dL, while other laboratory findings were within normal limits. Abdominal ultrasonography demonstrated increased echogenicity of the fatty tissue around the transverse colon and a contiguous hypoechoic lesion measuring 2.3 × 0.7 cm, suggestive of an abscess. A linear hyperechoic structure measuring approximately 1.0 cm was observed within the lesion and was suspected to represent a fish bone (**Fig. 1A**). Contrast-enhanced CT revealed inflammatory changes in the pericolic fat around the transverse colon and a contiguous abdominal wall abscess located ventral to the inflamed tissue. A linear calcified structure consistent with a foreign body was identified in the same region (**Fig. 1B**). No free air or ascites was detected. The patient reported a habitual intake of fish.

**Fig. 1:**
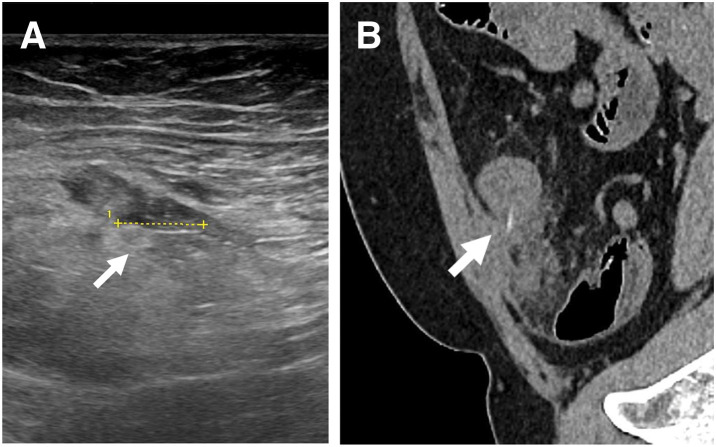
Initial imaging findings. (**A**) Abdominal ultrasonography demonstrating a linear hyperechoic structure corresponding to a retained fish bone (arrow). (**B**) Sagittal CT image showing an abdominal wall abscess adjacent to the transverse colon with a suspected fish bone (arrow).

Based on these findings, an abdominal wall abscess caused by penetration of the transverse colon by a fish bone was diagnosed, and the patient was admitted to our hospital. Because she showed no signs of peritoneal irritation and was hemodynamically stable, conservative treatment with intravenous cefmetazole (3 g/day) was administered for 10 days. Her abdominal pain improved rapidly, and she was discharged on hospital day 13.

Follow-up abdominal ultrasonography 1 month after discharge demonstrated a residual linear hyperechoic structure measuring 1.0 cm with a surrounding hypoechoic area measuring 2.4 × 0.3 cm at the same site. Follow-up CT showed marked shrinkage and near resolution of the abdominal wall abscess, while the fish bone remained identifiable (**Fig. 2**). Elective laparoscopic surgery was therefore planned. On the day before surgery, the site of the retained fish bone was marked on the abdominal wall under ultrasonographic guidance (**Fig. 3A** and **3B**).

**Fig. 2:**
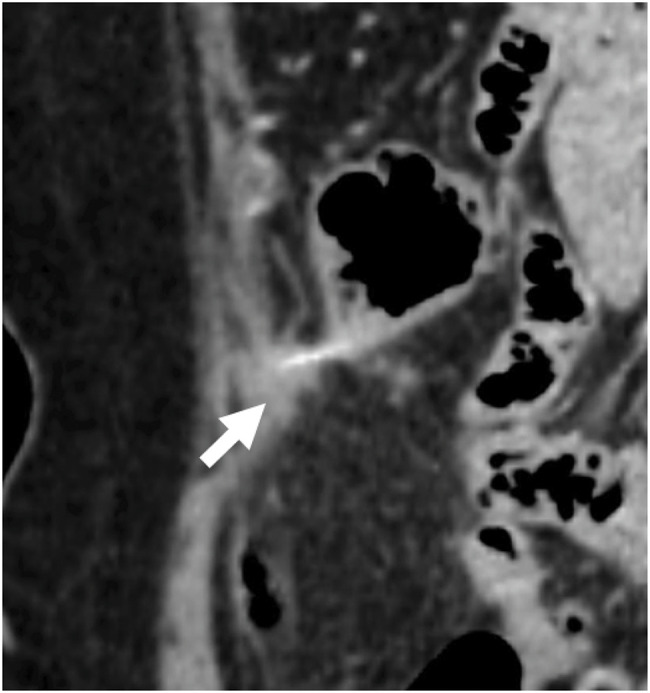
Follow-up CT after conservative treatment. Sagittal CT image demonstrating marked shrinkage of the abdominal wall abscess after conservative treatment, while a linear hyperdense foreign body consistent with a fish bone remains identifiable (arrow).

**Fig. 3:**
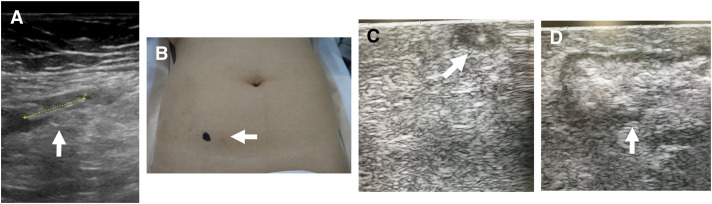
Ultrasonographic localization of the fish bone. (**A**) Preoperative abdominal ultrasonography demonstrating a retained fish bone in the right lower abdomen (arrow). (**B**) Preoperative ultrasonography-guided marking of the abdominal wall (arrow). (**C**) Intraoperative laparoscopic ultrasonography demonstrating a linear hyperechoic structure corresponding to the fish bone within the abdominal wall (arrow). (**D**) The fish bone is no longer visible, and the transverse colon is observed at a distance from the previous location of the fish bone (arrow).

Surgery was performed with the patient in the supine position under general anesthesia. A 12-mm camera port was placed at the umbilicus, a 12-mm working port in the left mid-abdomen, and a 5-mm port in the left lower abdomen. Intraoperatively, a small portion of the greater omentum was adherent to the abdominal wall beneath the preoperative marking (**Fig. 4**). A linear foreign body consistent with a fish bone was identified within the omentum. After adhesiolysis, the abdominal wall remained connected only to the fish bone and the adjacent intestinal tract. Laparoscopic ultrasonography was used to identify the precise location of the fish bone relative to the abdominal wall (**Fig. 3C**). The fish bone was found to be partially embedded in the abdominal wall, which was resected with a margin of surrounding granulation tissue to free the foreign body. Further dissection was performed to expose the transverse colon, and laparoscopic ultrasonography confirmed that the fish bone was not located within the colonic lumen (**Fig. 3D**). The fish bone, measuring approximately 1 cm in length, was successfully removed, and the procedure was completed. The postoperative course was uneventful.

**Fig. 4:**
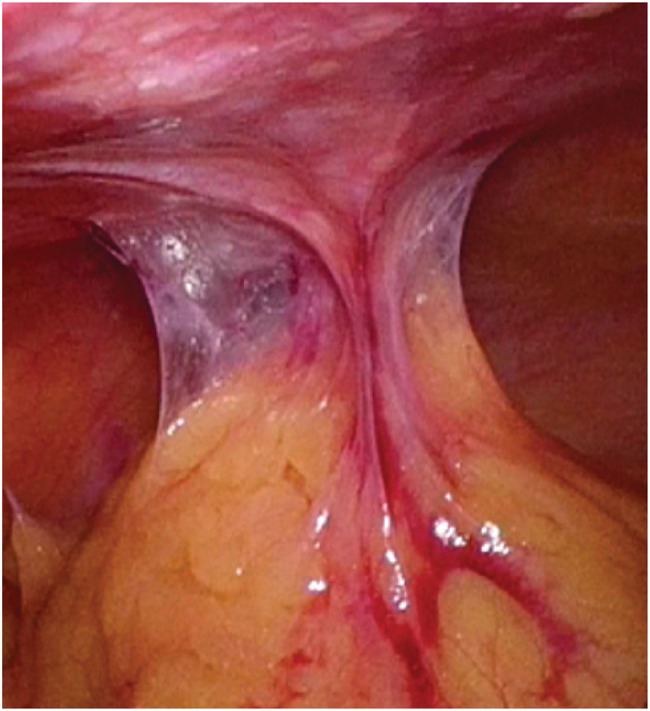
Intraoperative laparoscopic findings. Dense inflammatory adhesions between the abdominal wall and the adjacent intestine are observed, making direct visual identification of the foreign body difficult.

## DISCUSSION

The majority of ingested foreign bodies pass spontaneously through the gastrointestinal tract, and complications such as perforation occur in fewer than 1% of cases.^1)^ The types of foreign bodies differ by dietary habits; while toothpicks and chicken bones are more common in Western countries, fish bones are more frequently implicated in regions with high fish consumption.^1)^ Previous reports have indicated that fish bones account for a substantial proportion of gastrointestinal perforations caused by foreign bodies. Owing to their sharp tips and linear shape, fish bones are particularly prone to perforation and penetration of the gastrointestinal wall.^1)^ The ileum, transverse colon, and sigmoid colon have been reported as common sites of perforation. These segments are relatively mobile because they are not fixed to the retroperitoneum, which may predispose them to injury from peristaltic movement.^2)^ Although a history of accidental ingestion is important for diagnosis, many patients are unaware of ingestion, particularly in cases with a chronic clinical course, making preoperative diagnosis challenging.^3)^

CT is the most useful imaging modality for diagnosing fish bone perforation, typically demonstrating a linear hyperdense structure within the gastrointestinal tract or an associated abscess.^1)^ Multiplanar reconstruction using coronal and sagittal views can improve detection, with a reported sensitivity of up to 97%.^1)^ Abdominal ultrasonography is also useful, especially in the presence of an abscess, and in the present case, it clearly demonstrated a linear hyperechoic structure corresponding to the retained fish bone.^4)^

The pathological process of gastrointestinal perforation caused by foreign bodies can be broadly classified into acute and chronic inflammatory types.^1)^ Acute inflammation often results in generalized peritonitis and requires urgent intervention, whereas chronic inflammation progresses gradually, leading to localized abscess or granuloma formation.^1)^ Treatment strategies for chronic cases vary and may include conservative management, abscess drainage, endoscopic removal, or surgical intervention.^1)^ Although most reported cases are treated surgically, conservative treatment has been reported in a small proportion of patients.^5)^ However, several reports indicate that retained fish bones may lead to recurrent abscess formation, necessitating eventual removal.^2,3)^

In the present case, chronic inflammation without peritoneal signs allowed for initial conservative treatment with antibiotics, resulting in symptom resolution and shrinkage of the abscess. Elective laparoscopic surgery was subsequently performed. Although the patient was asymptomatic after conservative treatment, surgical removal of the retained fish bone was considered necessary due to the potential risk of recurrent abscess formation and chronic inflammation. Previous reports have indicated that retained foreign bodies may lead to delayed complications or recurrence.^2,3)^ In addition, the presence of a sharp foreign body outside the gastrointestinal tract carries a risk of further tissue injury or migration. Therefore, elective laparoscopic removal was selected as a definitive treatment. While expectant management may be considered in selected cases, definitive removal was considered a more reliable strategy in this case.

Laparoscopic surgery offers the advantages of minimal invasiveness and detailed intra-abdominal observation; however, identifying a small retained fish bone can be difficult due to adhesions and granulation tissue.^6–8)^ Preoperative body surface ultrasonography for localization and intraoperative laparoscopic ultrasonography were therefore employed. These techniques enabled accurate localization of the fish bone relative to the abdominal wall and transverse colon, allowing safe and minimally invasive removal without bowel resection.^4)^

In the present case, preoperative ultrasonographic marking was effective because the fish bone was partially embedded in the abdominal wall, which limited its mobility. However, when a foreign body is not fixed, such as when it is encapsulated within the omentum, the position may change due to pneumoperitoneum, patient positioning, or peristalsis. In such situations, intraoperative laparoscopic ultrasonography should be considered the primary modality for localization rather than an adjunct.

A laparoscopic ultrasonography probe was introduced through a 12-mm port and used to identify the fish bone as a linear hyperechoic structure within the abdominal wall. The spatial relationship between the foreign body and the transverse colon was clearly visualized, and dissection was performed under ultrasonographic guidance.

Laparoscopic management has been increasingly reported; however, intraoperative localization techniques are not routinely described, highlighting the practical value of laparoscopic ultrasonography in such cases.

This case demonstrates 2 important technical takeaways. First, staged management with initial conservative therapy can reduce inflammation and allow safer elective laparoscopic surgery in selected patients with chronic fish bone penetration.^5)^ Second, laparoscopic ultrasonography is a valuable adjunct for accurately localizing retained foreign bodies when visual identification is difficult. The novelty of this case lies in the practical intraoperative use of laparoscopic ultrasonography to enable precise localization and avoid bowel resection in a rare presentation complicated by an abdominal wall abscess.^1,9)^

## CONCLUSIONS

Staged management with initial conservative therapy followed by elective laparoscopic surgery enabled safe and minimally invasive removal of a retained fish bone causing an abdominal wall abscess. Laparoscopic ultrasonography was particularly useful for accurate localization of the foreign body when direct visual identification was difficult. This approach may be a valuable option in similar cases complicated by chronic inflammation.

## DECLARATIONS

### Use of artificial intelligence

The authors used an AI-based language tool to assist with English language editing and clarity. The authors take full responsibility for the content of the manuscript.

### Availability of data and materials

The data supporting the findings of this case report are not publicly available due to patient privacy concerns.
